# Identifying patterns and profiles of vaccination hesitancy among nurses for tailoring healthcare policies in the UK: A cross‐sectional study

**DOI:** 10.1111/inr.13035

**Published:** 2024-08-19

**Authors:** Goran Erfani, Jemma McCready, Bethany Nichol, Charlotte Gordon, John Unsworth, Michelle Croston, Dania Comparcini, Valentina Simonetti, Giancarlo Cicolini, Kristina Mikkonen, Jeremia Keisala, Marco Tomietto

**Affiliations:** ^1^ Department of Nursing, Midwifery and Health, Faculty of Health and Life Sciences Northumbria University Newcastle upon Tyne UK; ^2^ Department of Social Work, Education and Community Wellbeing, Faculty of Health and Life Sciences Northumbria University Newcastle upon Tyne UK; ^3^ Manchester University NHS Foundation Trust Manchester UK; ^4^ Department of Precision and Regenerative Medicine and Ionian Area University of Bari “Aldo Moro,” Bari Italy; ^5^ Department of Innovative Technologies in Medicine and Dentistry University “G. D'Annunzio” Chieti‐Pescara Chieti Italy; ^6^ Research Unit of Health Science and Technology Faculty of Medicine University of Oulu Oulu Finland

**Keywords:** COVID‐19, influenza, machine learning, nurses, profiles, vaccination campaigns, vaccination hesitancy

## Abstract

**Aims:**

To profile the characteristics of nurses with varying levels of vaccine hesitancy toward the COVID‐19 and influenza vaccines.

**Background:**

In many countries across the world, healthcare workers, and nurses in particular, display significant reluctance toward COVID‐19 and influenza vaccines due to concerns about safety, distrust in healthcare policies, and media influences. To address this, a proposed approach involves profiling nurses to tailor vaccination campaigns and to improve acceptance rates and public health outcomes.

**Methods:**

This cross‐sectional study adopted the Vaccination Attitudes Examination scale to assess hesitancy toward COVID‐19 and influenza vaccines among 294 registered nurses in the UK between March and July 2023. A K‐means cluster analysis was performed. The Strengthening the Reporting of Observational Studies in Epidemiology guidelines were adopted.

**Results:**

Three profiles were identified. Profile A showed low vaccination hesitancy, profile B showed average hesitancy, and profile C showed high hesitancy toward vaccines. The highest concern for all profiles was related to unforeseen future effects of vaccination. Profile C had more nurses in early career roles, whereas nurses in profiles A and B were in more senior roles. Profile A showed higher educational attainment. Nurses in profile C used Snapchat more, whereas nurses in profile A used Twitter more frequently.

**Conclusion:**

This study identified specific characteristics associated with higher levels of vaccination hesitancy in nursing. Unforeseen future effects of vaccination are a core aspect to consider in promoting vaccination.

**Implications for nursing and nursing policy:**

Policies and vaccination campaigns should be targeted on early career nurses and should deliver tailored messages to dispel misinformation about unforeseen future effects of vaccination through specific social media platforms. Senior nurses should be involved as role models in promoting vaccination. These results are key for enhancing an evidence‐based approach to implementing global health policies in healthcare.

## INTRODUCTION

Vaccine hesitancy is recognized as one of the top 10 challenges to global well‐being, and it is defined as the reluctance or resistance to receiving vaccinations, even when vaccines are accessible (WHO, 2019a). The reasons behind this hesitancy are complex, influenced by an individual's perceptions and attitudes toward specific vaccines or immunizations in general, as well as their sociodemographic attributes (such as gender and ethnicity) and contextual elements (such as trust in experts and perceived risks) (Larson et al., 2022). Addressing vaccine hesitancy among healthcare workers is a significant policy imperative because of the occupational risk of infection, the risks to immunocompromised and vulnerable patients, and the challenges associated with workforce availability (Maltezou et al., 2022). From a policy perspective, there is a tension between mandatory and voluntary vaccination, with some countries stepping back from making the COVID‐19 vaccine a condition of deployment because of the potential loss of a significant number of staff (Gravagna et al., 2020). In terms of vaccine policy, several countries including the United States, New Zealand, Poland, France, Greece, and Italy moved to a policy of mandatory vaccination against COVID‐19 for healthcare workers, although many have subsequently relaxed the requirements (Wise, 2021). Other countries have mandatory vaccine requirements related to initial employment and then adopt a voluntary system for annual vaccines (Bianchi et al., 2022; WHO, 2022). The World Health Organization (2019b) has outlined the need for further research to understand the perceptions, motivators, and barriers to vaccine acceptance among healthcare workers.

The significance of understanding the drivers of vaccine hesitancy has become more evident, especially among HCWs and nurses in particular. The global prevalence of vaccination hesitancy among over 75,000 HCWs revealed that one‐fifth of these professionals were reluctant to accept a COVID‐19 vaccine (Biswas et al., 2021). Also, the seasonal influenza vaccination uptake among HCWs in European countries from 2015 to 2018 was less than 40%, far behind the expected target of 75% (European Centre for Disease Prevention & Control, 2018). The levels of hesitation among HCWs raise significant concerns for several reasons: (1) HCWs face an elevated risk of contracting the virus, potentially leading to increased staff shortages at a time when healthcare services are in higher demand (Grochowska et al., 2021); (2) HCWs are more prone to becoming carriers of the virus, transmitting infections to clinically vulnerable individuals (Asad et al., 2020); and (3) recommendations for vaccination from HCWs have been proven to positively influence vaccine acceptance among the general population (McCready et al., 2023).

Among the HCWs population, nurses showed higher vaccination hesitancy than other occupational roles, with nurses’ vaccination hesitancy being 15% to 50% higher compared with other HCWs (McCready et al., 2023). Vaccination hesitancy within the nursing profession is a complex phenomenon. Some core factors are concerns about vaccine‐related information, lack of evidence‐based data on vaccine safety, mistrust in the government or healthcare system's policies, and concerns related to the economic benefits to pharmaceutical companies (Khubchandani et al., 2022). Other factors, such as younger age and identifying with a female gender, are also related to higher vaccination hesitancy in nursing populations (Yasmin et al., 2021). Additionally, vaccination hesitancy is influenced by information distributed by traditional media and social media platforms (Dini et al., 2018). Currently, the interaction between these different components in disclosing vaccination hesitancy, especially among nurses, is not clear. Identifying specific profiles of vaccination hesitancy and defining the characteristics of those profiles is key to further addressing vaccination policies and improving patient safety, global health, and public health outcomes.

Globally, a range of approaches to increase the uptake of influenza vaccine among healthcare workers have been evaluated. Approaches fall into four broad categories, with most providers concentrating on tailored communication. The WHO (2019b) recommends that communication concentrates on the benefit to the individual and details how to access the vaccine. The evidence base behind such communications is derived from the patient facing communication rather than communicating directly to healthcare workers (Public Health Wales, 2023). Some providers have developed approaches to vaccination availability by scheduling appointments and including these in the communication, whereas others have made mobile carts available (Stead et al., 2019). Immunization campaigns often focus on promotional materials or the communication of simple measures. Such campaigns have been shown to be useful to increase uptake in hospitals with no prior immunization policy (Heinrich‐Morrison et al., 2015). Finally, nudges have been used to reinforce messages and can include a variety of messages about case rates and outbreaks and to communicate reminders about pre‐set appointments (Stead et al., 2019).

To identify potential approaches to improving vaccination uptake, it is critical to understand the diverse patterns and the interplay of various characteristics that contribute to vaccination hesitancy for registered nurses. This study aims to profile the characteristics of nurses to determine factors associated with vaccination hesitancy by adopting a clustering technique. This understanding will assist healthcare organizations and policymakers in developing future vaccination campaigns and policies to promote vaccination.

## METHOD

### Design and sample

This study employed a cross‐sectional design through an online survey between March and July 2023. A convenience sampling approach was adopted. The Strengthening the Reporting of Observational Studies in Epidemiology (STROBE) guidelines were adopted. The inclusion criteria for participating in the survey were being a registered nurse and providing informed consent. No specific inclusion criteria were required to run the data analysis adopted in this study and described below. The sample size estimation was calculated in G*Power v3.1 (Faul et al., 2009) by considering three clusters, an alpha error of 0.05, a power of 0.95, and an effect size of 0.25: a total sample of 252 participants was considered adequate. A final sample of 294 participants was recruited via an online survey. The survey was disseminated through formal and informal networks of nurses, social media, professional groups and associations, and organizational newsletters. Both hospital and community healthcare settings in the center and north‐east of England were involved.

### Variables and instruments

The Vaccination Attitudes Examination (VAX) scale was adopted to assess vaccination hesitancy regarding COVID‐19 and influenza vaccines. This scale is internationally adopted to measure vaccination hesitancy, and it comprises 12 items rated on a Likert scale ranging from one (totally disagree) to seven (totally agree) (Martin & Petrie, 2017). The factors of the VAX scale included “mistrust of vaccine benefit” (3 items), “worries about unforeseen future effects” (3 items), “concerns about commercial profiteering” (3 items), and “preference for natural immunity” (3 items). Lower scores reflected a more positive attitude toward the vaccine. The VAX scale is available in the public domain and adopted internationally in public health. Sociodemographic, work‐related (i.e., work or placement in a COVID area), health‐related (COVID‐19 infection exposure), and media and social media usage data (frequency of use of different TV and radio channels and social media platforms scored from never (1) to always (4)) were also collected to describe the sample and compare the clusters’ characteristics.

### Data analysis

The data were analyzed using SPSS v28 (IBM Corp., 2021). K‐means cluster analysis was performed to detect the clusters of vaccine hesitancy. K‐means cluster analysis is an unsupervised machine learning approach useful to identify the underlined patterns and describe those patterns (Ikotun et al., 2023). The data standardization was unnecessary because the raw data were measured in the same units for all variables, and the VAX factor scores ranged from 1 to 7. The optimal number of clusters was identified by performing a two‐step cluster analysis with silhouette measures of cohesion by considering the Euclidean distance metric. In this study, the K‐means clustering detected three distinct clusters. The silhouette measure of cohesion and separation for a three‐cluster solution was 0.6, indicating a “good solution.” This approach allowed the employing of an iterative algorithm to minimize within‐cluster and maximize between‐cluster distances to confirm the three‐cluster solution (Wu, 2012). The factors of the VAX scale were considered to identify the K‐means clusters and the data points to define them. A maximum of 50 iterations were set to achieve the model's convergence and the convergence criterion was set at 0. In this study, 16 iterations were necessary to achieve convergence. The Forgy method was adopted for initializing cluster centroids; this method is considered computationally efficient, and it randomly selects k observations from the data set to use their values as initial cluster centroids. As multivariate outliers tend to jeopardize the K‐means cluster analysis and produce suboptimal clustering results (Ikotun et al., 2023), their presence was tested by calculating the Mahalanobis distances and the *p*‐value of the chi‐square distribution, considering 24 degrees of freedom. No outliers were detected in the data distribution. The expectation maximization algorithm was used to manage the K‐means clustering computation. One‐way ANOVA and chi‐square tests were adopted to assess the statistical significance of the differences among clusters. A *p*‐value < 0.05 indicated an adequate statistical significance.

### Validity, reliability, and rigor

To ensure content validity, a panel of eight experts rated both scales for relevance and clarity, and the content validity index was calculated. The experts were identified in order to cover a range of expertise from public health, research, clinical practice, and leadership, by adopting a purposive sampling criterion. The cutoff for the content validity index (I‐CVI) value was set at 0.83 at the scale level (S‐CVI) (Polit & Beck, 2006). In this study, relevance and clarity of the VAX scale were, respectively, 0.86 and 0.88.

Cronbach's alpha was calculated for each factor of the VAX scale to assess reliability for both the Influenza and COVID‐19 scales. Values ≥ 0 .70 are considered as acceptable (DeVellis, 2016).

In this study, the Cronbach's alpha for the VAX scale related to Influenza vaccine hesitancy ranged from 0.76 to 0.97 among the four factors, whereas the Cronbach's alpha of the VAX scale for the COVID‐19 vaccine ranged from 0.85 to 0.97.

### Ethical considerations

Data collection and analysis procedures were designed to ensure data confidentiality and alignment with both national and European laws, including the General Data Protection Regulations (GDPR) and the UK Data Protection Act (Carey, 2018). By submitting the survey, participants provided their consent to participate in the study. Ethical approval was granted by Northumbria University (ref: 2948, date: February 27, 2023).

## RESULTS

### Characteristics of the participants

The respondents had an average age of 48.26 years (SD = 10.37), and most respondents were female (86.4%). Respondents reported an average work experience of 11.37 years (SD = 9.42) in their current area of clinical practice. The average work experience in the nursing profession (years post qualification) was 24 years (SD = 12.15). Reported highest academic qualifications were as follows: diploma (*n* = 24; 8.2%); advanced diploma (*n* = 21; 7.1%), BSc (*n* = 19; 6.5%), BSc (Hons) (*n* = 90; 30.6%), MSc (*n* = 112; 38.1%), and PhD (*n* = 28; 9.5%).

### Vaccination hesitancy profiles

Nurses clustered in profile A had the lowest mean scores for each of the VAX factors than nurses in the other two clusters. Therefore, profiles A, B and C could be categorized as low vaccination hesitancy (COVID‐19: mean = 1.97, SD = 0.47; influenza: mean = 1.92, SD = 0.43), average vaccination hesitancy (COVID‐19: mean = 3.25, SD = 0.49; influenza: mean = 3.03, SD = 0.47), and high vaccination hesitancy (COVID‐19: mean = 5.56, SD = 0.58; influenza: mean = 4.93, SD = 0.75), respectively. Nurses clustered in each profile reported their “worries about unforeseen future effects” as the highest aspect of vaccination hesitancy against COVID‐19 (profile A: mean = 3.15, SD = 1.09; profile B: mean = 4.55, SD = 1.03; profile C: mean = 6.20, SD = 0.86) and influenza (profile A: mean = 2.98, SD = 1.02; profile B: mean = 4.26, SD = 1.06; profile C: mean = 5.53, SD = 0.91). Nurses clustered in profile A were least hesitant due to “concerns about commercial profiteering of COVID‐19” (mean = 1.44, SD = 0.59), while nurses in profile B scored their “mistrust of vaccine benefits for Influenza” the lowest (mean = 2.15, SD = 0.77) and nurses in profile C reported the lowest score for “concerns over commercial profiteering of Influenza vaccine” (mean = 4.67, SD = 1.19) as their most positive attitudes toward vaccination (Table 1).

**TABLE 1:** Descriptive statistics for vaccine hesitancy scores (VAX) toward COVID‐19 and influenza vaccines for the three profile clusters.

VAX factors	Profile A (*n* = 162) Mean (SD)	Profile B (*n* = 93) Mean (SD)	Profile C (*n* = 39) Mean (SD)	*F* ^a^	*P* value
COVID‐19					
Mistrust of vaccine benefit	1.75 (0.91)	2.42 (0.89)	5.37 (1.33)	220.23	**<0.001**
Worries about unforeseen future effects	3.15 (1.09)	4.55 (1.03)	6.20 (0.86)	153.38	**<0.001**
Concerns about commercial profiteering	1.44 (0.59)	2.61 (0.92)	5.35 (1.32)	357.58	**<0.001**
Preference for natural immunity	1.56 (0.66)	3.42 (0.94)	5.33 (1.02)	404.23	**<0.001**
Influenza					
Mistrust of vaccine benefit	1.65 (0.87)	2.15 (0.77)	4.80 (1.443)	180.54	**<0.001**
Worries about unforeseen future effects	2.98 (1.02)	4.26 (1.06)	5.53 (0.91)	118.20	**<0.001**
Concerns about commercial profiteering	1.45 (0.53)	2.54 (0.87)	4.67 (1.19)	289.61	**<0.001**
Preference for natural immunity	1.60 (0.65)	3.17 (0.87)	4.70 (1.06)	290.92	**<0.001**

*Note*. The mean difference is statistically significant at *p* < 0.001 (highlighted in bold). The vaccination hesitancy score was based on a seven‐point Likert scale (scores 1−7).

^a^ One‐way ANOVA *F* test, including multiple pairwise comparisons conducted with Bonferroni correction; each comparison demonstrated a *p*‐value < 0.001.

### Profiling of sociodemographic characteristics

There was a statistically significant difference (*p *= 0.005) in the ages of nurses who clustered in profile A (mean = 48.99, SD = 10.13) and profile C (mean = 43.23, SD = 11.03) (Table 2). In terms of role, profile C was represented by nurses who held a position in clinical practice (band 5 and 6 positions in the UK) (60%), whereas profiles A and B were represented by nurses in senior leadership roles (band 7 or higher in the UK). The nurses clustered in profile B reported longer work experience in the nursing profession (mean = 25.54 years, SD = 11.20 years). Nurses clustered in profile C reported the least extensive work experience in nursing (mean = 17.00 years, SD = 13.10). Regarding the highest academic award, profile A was represented by nurses with postgraduate degrees (56%), whereas profiles B and C were represented by nurses with undergraduate degrees (45.2% and 56.4%, respectively).

**TABLE 2:** Nurse (*n* = 294) characteristics, based on their distribution to profiles A, B, and C.

Characteristics	Profile A (*n* = 162)	Profile B (*n* = 93)	Profile C (*n* = 39)	*F* ^a^/*X* ^2^ ^b^	*p*‐value
Ages in years, mean (SD)	48.99 (10.13)	49.10 (10.03)	43.23 (11.03)	*F *= 5.45	**0.005** ^*^
Gender, *n* (%)					
Female	137 (84.6)	81 (87.1)	36 (92.3)	*X^2 ^* = 4.194	0.650
Male	23 (14.2)	10 (10.8)	3 (7.7)		
Missing values	2 (1.2)	2 (2.2)			
Role, *n* (%)					
Registered nurse (band 5)	20 (12.3)	11 (11.8)	11 (28.2)	*X^2 ^* = 30.695	**<0.001**
Nurse specialist (band 6)	22 (13.6)	13 (14.0)	12 (30.8)		
Ward manager (band 7)	41 (25.3)	17 (18.3)	8 (20.5)		
Chief nurse (band 8)	37 (22.8)	25 (26.9)	2 (5.1)		
Nurse educator	18 (11.1)	20 (21.5)	5 (12.8)		
Other	24 (14.8)	7 (7.5)	1 (2.6)		
Areas of practice, *n* (%)					
Community	61 (37.7)	32 (34.4)	14 (35.9)	*X^2 ^* = 0.274	0.872
Hospital settings	101 (62.3)	61 (65.6)	25 (64.1)		
Field of nursing practice, *n* (%)					
Adult	122 (75.3)	75 (80.6)	32 (82.1)	*X^2 ^* = 10.682	0.172
Child	11 (6.8)	7 (7.5)	2 (5.1)		
Mental Health	8 (4.9)	3 (3.2)	5 (12.8)		
Learning disabilities	2 (1.2)	1 (1.1)	0 (0.0)		
Other	19 (11.7)	7 (7.5)	0 (0.0)		
Work experience in years, mean (SD)	11.44 (9.36)	12.44 (9.53)	8.49 (9.03)	*F *= 2.456	0.088
Worked in nursing (years post qualification), mean (SD)	24.90 (11.94)	25.54 (11.20)	17.00 (13.10)	*F *= 8.022	**<0.001** ^*^
Highest academic award, *n* (%)					
Diploma/advanced diploma	26 (16.0)	12 (12.9)	7 (17.9)	*X^2 ^* = 17.013	**0.002**
BSc/BSc (Hon)	45 (27.8)	42 (45.2)	22 (56.4)		
MSc/PhD	91 (56.2)	39 (41.9)	10 (25.6)		
Social media apps, mean (SD)	(*n* = 131)	(*n* = 77)	(*n* = 24)		
Facebook	2.95 (1.07)	3.08 (1.05)	2.92 (1.06)	*F *= 0.394	0.675
YouTube	2.36 (0.85)	2.26 (0.70)	2.38 (0.647)	*F *= 0.459	0.632
WhatsApp	3.49 (0.762)	3.46 (0.93)	3.53 (0.60)	*F *= 0.131	0.877
Instagram	2.29 (1.12)	2.55 (1.20)	2.50 (1.22)	*F *= 1.290	0.277
TikTok	1.31 (0.72)	1.35 (0.66)	1.38 (0.72)	*F *= 0.118	0.889
Snapchat	1.16 (0.44)	1.25 (0.65)	1.63 (1.01)	*F *= 6.164	**0.002**
Pinterest	1.50 (0.70)	1.61 (.63)	1.54 (0.83)	*F *= 0.518	0.596
Reddit	1.19 (0.56)	1.01 (0.11)	1.09 (0.29)	*F *= 4.142	**0.017**
LinkedIn	1.78 (0.95)	1.61 (0.85)	1.38 (0.71)	*F *= 2.434	0.090
Twitter (now X)	2.90 (1.20)	2.38 (1.04)	1.96 (0.10)	*F *= 9.925	**<0.001**
Broadcasters, mean (SD)	(*n* = 131)	(*n* = 77)	(*n* = 24)		
Public service	2.60 (0.73)	2.90 (0.78)	2.42 (0.84)	*F = 5.157*	**0.006**
Opinion‐orientated	1.06 (0.22)	1.01 (0.06)	1.21 (0.49)	*F = 7.010*	**0.001**
Get vaccinated for influenza, *n* (%)				*X^2 ^* = 103.723	**<0.001**
Yes	154 (95.1)	86 (92.5)	8 (20.5)		
No	2 (1.2)	4 (4.3)	19 (48.7)		
Not every year	6 (3.7)	3 (3.2)	12 (30.8)		
Been affected by COVID, *n* (%)				*X^2 ^* = 2.655	0.858
No	24 (14.8)	12 (12.9)	5 (12.8)		
Once	83 (51.2)	50 (53.8)	18 (46.2)		
Twice	37 (22.8)	17 (18.3)	11 (28.2)		
More than twice	18 (11.1)	14 (15.1)	5 (12.8)		
Vaccinated against COVID *n* (%)				*X^2 ^* = 55.646	**<0.001**
Yes	158 (97.5)	85 (91.4)	19 (48.7)		
No	4 (2.5)	8 (8.6)	20 (51.3)		

*Note*. The mean difference is statistically significant at *p* < 0.05 (highlighted in bold). Percentages may not add to 100% due to rounding.

^a^ One‐way ANOVA *F* test, including multiple pairwise comparisons conducted with Bonferroni correction.

^b^ Chi‐square test and Fisher exact test were performed if the expected frequency of cells was less than 20%.

* Clusters A and C differed significantly in age (*p* < 0.005) and working in nursing (*p* < 0.001) variables based on the one‐way ANOVA *F* test, including multiple comparisons with Bonferroni correction.

### Profiling of media exposure

In relation to the use of social media applications, nurses in profile C, on average, had a significantly higher level of using Snapchat (mean = 1.63, SD = 1.01) compared with the nurses in profile A (mean = 1.16, SD = 0.44) and nurses in profile B (mean = 1.25, SD = 0.65). However, nurses in profile C reported a lower level of using Twitter (mean = 1.96, SD = 0.10) than nurses in profile A (mean = 2.90, SD = 1.2). Nurses clustered in profile B (mean = 1.01, SD = 0.11) reported a lower level of using Reddit than nurses in profile A (mean = 1.19, SD = 0.56). Nurses in profiles A and B tended to watch and listen to public service broadcasters (*F *= 5.157, *p* = 0.006), whereas nurses clustered in profile C tended to watch and listen to the opinion‐orientated broadcasters (*F *= 7.010, *p* = 0.001) (Table 2).

### Profiling of current vaccination behaviors

More than 90% of nurses clustered in profiles A and B reported getting vaccinated for influenza every year, but nurses in profile C mostly reported “no” or “not every year” (*X* ^2 ^= 103.723, *p* < 0.001). Similarly, more than 90% of nurses clustered in profiles A and B were fully vaccinated against COVID‐19, whereas more than half of nurses in profile C were not (*X* ^2 ^= 55.646, *p* < 0.001) (Table 2).

## DISCUSSION

This study aimed to identify factors of vaccination hesitancy among registered nurses and identify distinct nurses' profiles by performing a cluster analysis. The results demonstrated that vaccination hesitancy among nurses is largely a result of anxiety about unforeseen future effects, and this applies to both COVID‐19 and influenza vaccines. This finding aligns with previous studies that highlighted the strong influence of vaccine safety as the primary determinant of vaccine hesitancy among HCWs (Li et al., 2021), including nurses from different generations (Tomietto et al., 2022). The literature links this issue to misinformation (and disinformation) spread on social media platforms and insufficient information/evidence about vaccine benefits or potential risks (Krishnan et al., 2021). Evidence‐based information on vaccine safety and consistency in disseminating the information among nurses should be a top policy priority.

The results showed that age, academic degree, work experience, and roles in the nursing profession were associated with vaccination hesitancy for both influenza and COVID‐19. In detail, older nurses, with higher educational attainment and greater years of service in the nursing profession, had significantly lower levels of vaccine hesitancy toward both vaccinations compared with younger, early‐career nurses. These results suggest that as nursing staff take on more managerial and team leadership responsibilities, they are more likely to be vaccinated. This is a significant new finding, especially from the angle of those vaccinated against influenza and COVID‐19 but who still exhibit slightly hesitant attitudes (profile B). This finding is also significant when considering the policy approaches to improving vaccination uptake. Although many organizations have vaccine campaigns, especially around the influenza vaccine, they often use the nursing and medical directors as role models for vaccination (Heinrich‐Morrison et al., 2015). This research suggests that direct peers and immediate line managers may be more effective as they have greater vaccine uptake despite some degree of vaccine hesitancy. This result is consistent with other recent studies that found a similar higher uptake among nursing supervisors than registered nurses (Tamburrano et al., 2019)—who typically are younger, less educated nurses with lower professional titles and fewer years of nursing service (Zhang et al., 2022). From a policy perspective, using more experienced supervisors and team leaders to promote vaccination among staff would be an effective intervention. For instance, mentors and supervisors are best placed to provide support to junior nurses and can positively influence nurses’ attitudes toward vaccination. These findings support the International Council of Nurses’ statements and policy regarding vaccination as a professional responsibility for nurses, as they act as role models and key sources of health advice (ICN, 2022; Kennedy, 2021).

Regarding social media apps, the results showed new patterns linked to vaccination hesitancy. Specifically, using Snapchat was associated with higher scores for vaccination hesitancy among nurses. In contrast, using Reddit and Twitter was associated with lower vaccination hesitancy. Despite Snapchat and Reddit's partnership with the UK Government (2021) to promote vaccination uptake in users, our results indicate that these platforms can have varying effects on the vaccination intentions of nurses. A recent study (Krishan et al., 2021) demonstrated that Twitter (now X), at the time, prohibited “misinformation regarding the nature of the virus, the efficacy and safety of prevention and treatment measures, COVID‐19 vaccines, and restrictions and health advisories, as well as content that misrepresented data” (Krishan et al., 2021). However, Snapchat and Reddit “did not articulate the specific types of COVID‐19 content they prohibited” (Krishan et al., 2021). As such, variations in prohibited content, criteria guiding responses, and strategies to address misinformation (Krishan et al., 2021) suggest the need to articulate more cohesive enforcement of regulations and consistent policies across social media platforms to address vaccination misinformation. Continuation of this inconsistency and variations could negatively affect the younger generations, including younger nurses, who more often use social media, specifically Snapchat. With increasing age, the frequency of using social media and, consequently, their influence is reduced (Lefebvre et al., 2020).

The results showed that the frequency of watching public service broadcasters was significantly higher among nurses associated with lower vaccination hesitancy (profiles A and B). In contrast, nurses associated with the highest vaccination hesitancy (profile C) more often relied on opinion‐orientated broadcasters. This is an important and new finding as public service broadcasters generally have a more balanced approach in terms of information on vaccination. Several opinion‐orientated broadcasters have been subject to investigation and sanctions by the Office of Communications in the UK during the pandemic (Ofcom, 2023), and these results empirically corroborate the need for a policy regulation regarding media and public health at the international level. Adherence to the standards of regulation for television and radio would ensure balance in reporting the benefits and potential harms in relation to vaccination.

### Implication for nursing and health policy

Based on the results of this study, several recommendations for nursing and policy can be identified. In particular, intervention strategies should consider utilizing nurses in leadership positions to deliver vaccination campaigns within healthcare settings. As such, senior nurses are well placed to address the concerns of younger, early career nurses, especially around concerns about unforeseen future effects of the vaccines. It is reasonable to suggest, based on our findings, that addressing concerns around the safety, effectiveness, and side effects of vaccines will reduce overall hesitancy and lead to more positive behaviors around vaccine uptake across the individual's nursing career. Moreover, the media and social media patterns identified in this study can inform additional strategies to boost vaccination rates in nurses and should be taken into consideration when designing government actions and policies for improving vaccination uptake across health and social care settings. Such findings may inform policies and regulations for mitigating the spread of information on social media platforms contributing to vaccination hesitancy. These results are consistent with the global nursing public health priorities and provide an evidence‐based foundation for policy development in this area. This study specifically supports the ICN statements regarding vaccination as a professional responsibility for nurses and it provides key insights into healthcare systems’ policy regarding vaccination campaigns along with media communication policies on this topic.

### Limitations

Although this study has found significant results, several limitations should be considered when interpreting our results. First, a large proportion of the sample was recruited from the North East of England—an area associated with poorer socioeconomic status (SES)—where vaccine uptake is typically lower than in more affluent areas of the UK (e.g., South East of England) (GOV.UK, 2023). On the other hand, a strength is that the profiling characteristics could be used for developing interventions tailored to nurses in regions with lower SES, ultimately improving vaccine uptake rates in these areas, which are a core priority in public health. This study assessed attitudes toward vaccine‐related factors (e.g., safety and benefits, commercial profiteering), and other factors contributing to vaccination hesitancy were not investigated. For instance, poor health status, low‐risk perceptions, and lack of trust in the government and local health authorities all contribute to vaccine hesitancy in HCWs (McCready et al., 2023). As such, there is a possibility that these factors might be important in identifying clusters of vaccine‐hesitant nurses. However, it may be that only assessing attitudes toward vaccination could potentially suffice, thus saving time and resources when identifying nurses in the workforce to engage in interventions. Furthermore, this study did not investigate the nature of the content that participants were exposed to on the social media platforms they regularly utilized. This gap in understanding limits the depth of analysis, and future research should explore this further. Lastly, although most respondents answered optional questions related to social media platforms and TV/radio channels, the sample size for these two categories was reduced. This resulted in slightly different sample sizes for each profile when comparing social media‐related characteristics. In addition, TV/radio channels and their contents are often endorsed on social media networks, for example, when individuals encounter a given channel content being widely shared on Twitter or other apps. The analysis recognizes and acknowledges the potential impact of this exchange.

## CONCLUSION

This study identified the key characteristics and attitudes of nurses associated with varying levels of hesitancy toward COVID‐19 and influenza vaccines. The profiles identified provide support for using a targeted approach to specific characteristics of nurses to tackle vaccination hesitancy. In detail, it is key to prioritize the dissemination of evidence‐based information on vaccine safety and address concerns about unforeseen future effects of vaccination. Moreover, it is important to target evidence‐based information to early‐career nurses through media and social media channels. Senior nurses in leadership responsibilities should be supported as role models to promote vaccination in healthcare settings. Collaboration with professional organizations, education and training, and engagement with media channels are key to implementing vaccination promotion initiatives and providing accurate and unbiased information. Together, the findings of this study lend support for an evidence‐based approach to nursing leadership for implementing global health policies in healthcare.

## AUTHOR CONTRIBUTIONS

*Study design*: Marco Tomietto, Giancarlo Cicolini, and Kristina Mikkonen. *Data collection*: Bethany Nichol, Jemma Mccready, Dania Comparcini, Valentina Simonetti, Jeremia Keisala, John Unsworth, Charlotte Gordon, and Michelle Croston. *Data analysis*: Goran Erfani and Marco Tomietto. *Study supervision*: Marco Tomietto and John Unsworth. *Manuscript writing*: Bethany Nichol, Jemma Mccready, Dania Comparcini, Valentina Simonetti, and Jeremia Keisala. *Critical revisions for important intellectual content*: Marco Tomietto, Kristina Mikkonen, Charlotte Gordon, John Unsworth, and Michelle Croston.

## CONFLICT OF INTEREST STATEMENT

The authors have no conflicts of interest to declare.

## ETHICAL APPROVAL

Ethical approval was granted by the Northumbria University Ethics Committee (ref: 2948, Feb. 27, 2023) and all participants provided informed consent before participating in this study.
